# An activity concentration-based proposal for radon management in workplaces involving NORM in Canada

**DOI:** 10.1007/s00411-024-01100-4

**Published:** 2024-12-18

**Authors:** Jing Chen, Debora Quayle

**Affiliations:** Radiation Protection Bureau, Health Canada, 775 Brookfield Road, Ottawa, ON K1A 1C1 Canada

**Keywords:** Rn-222, Radon management, NORM industry

## Abstract

The *Canadian Guidelines for the Management of Naturally Occurring Radioactive Materials* (NORM) have been developed to manage radiation doses received in workplaces involving NORM, such as mineral extraction and processing, oil and gas production, metal recycling or water treatment facilities. This management strategy works well for most naturally occurring radioactive materials in workplaces, with the exception of radon. Radon is a naturally occurring radioactive gas generated by the decay of uranium-bearing minerals in rocks and soils. Because radon exists everywhere in varying concentrations, it is not feasible to use incremental radon generated or enhanced by a practice as a means for assessing the need for radon management programs. Drawing from lessons learned through implementing the current NORM Guidelines, we propose decoupling the decision thresholds for NORM management (excluding radon) and radon management so that the two are considered separately, and quantifying decision-points for managing occupational radon exposure as average annual activity concentrations, with no requirement for dose calculations. Proposed application of this approach in the updated Canadian NORM Guidelines is described.

## Introduction

Radon is a naturally occurring radioactive gas generated by the decay of uranium-bearing minerals in rocks and soils. Radon exists everywhere in varying concentrations. Since radon is a gas, it can move freely through the soil, enabling it to escape into the atmosphere or seep into the indoor environment. Epidemiological studies have confirmed that exposure to radon increases the risk of developing lung cancer. Exposure to indoor radon has been determined to be the second leading cause of lung cancer after tobacco smoking (WHO 2009).

Authorities in Canada try to manage radon exposure through a number of mechanisms, including education campaigns, public and occupational health policy, and regulation. Canada has established a national guideline of 200 Bq/m^3^, average annual exposure, which is still voluntary in many situations but is gradually being adopted into regulation for schools, daycares, and workplaces. For workplaces that fall under Canadian nuclear legislation, including uranium mines and mills, radon exposure for workers is included in total occupational exposure and managed accordingly.

Canada has also developed the *Canadian Guidelines for the Management of Naturally Occurring Radioactive Materials* (the NORM Guidelines) (Health Canada 2011), specifically for managing radiation exposure caused by industries that are not licensed nuclear facilities but are engaging in activities where there is a risk that occupational or off-site public doses from NORM could exceed 1 mSv/a. For the remainder of this paper, these will be referred to as “NORM workplaces.” Non-uranium mining, mineral extraction and processing, oil and gas production, metal recycling, water treatment facilities, and fish hatcheries all fall within the category of NORM workplaces. Employees in these NORM workplaces are not classified as nuclear workers. Rather, they fall within the jurisdiction of Canadian provincial and territorial governments, with exposure management typically included in occupational health and safety legislation. It is expected that NORM Management (Table 1) is reasonably achievable for most Canadian NORM workplaces.

The current version of the NORM Guidelines has established thresholds for making decisions on worksite NORM management requirements, ostensibly based on the sum of the incremental doses from the work practice and total dose from radon, in the same manner as occupational exposure is calculated for licensed facilities. These thresholds and the corresponding requirements are described in Table 1.

**Table 1:** Dose thresholds in the Canadian NORM guidelines at which measures to manage worker exposure are recommended (Limits to manage public exposure exist but are not included here)

Threshold, E^a^, (mSv/a)	Classification	Expected actions (workers)
< 1	NORM Management	• Unrestricted for workers • (Public access restrictions may apply)
> 1 but < 5	Dose Management	• Public access restrictions • Measures to enable workers to better manage exposure, including signage, training, dosimetry (measured or estimated) with results reported to the national registry • Consider reviewing procedures, practices, and engineering controls to further optimize
> 5	Radiation Protection Management	As for Dose Management, and • Formal radiation protection program, consistent with requirements for licensed nuclear facilities • Dosimetry provided by a licensed dosimetry service

^a^: Annual projected effective dose

**Fig. 1:**
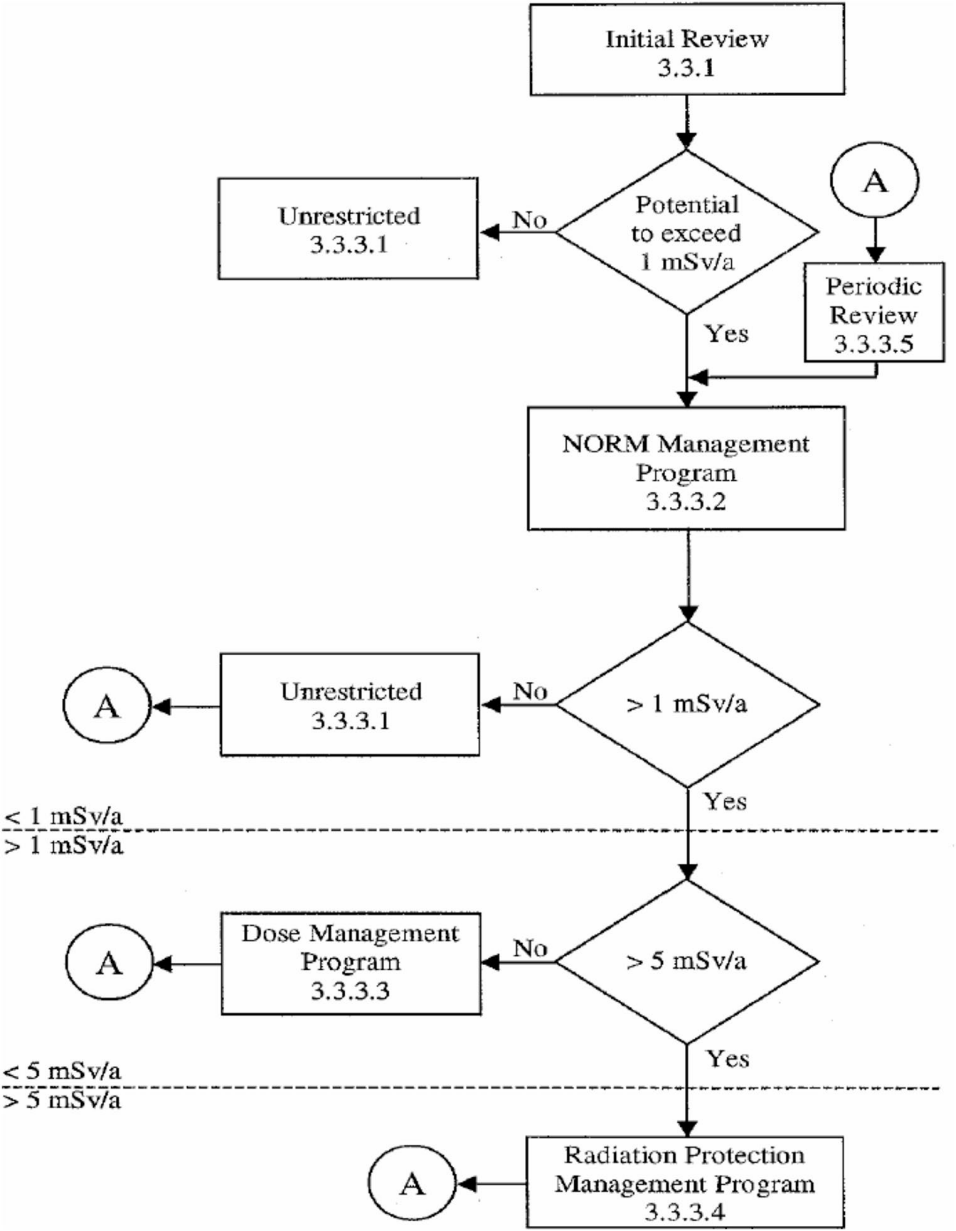
NORM management flowchart for workers. The circled A is the connection point to *Periodic Review*

In order to help users navigate the process of determining the scale of program required (or if a program is required at all), the Canadian NORM Guidelines also provide a flowchart for decision-making (Fig. 1). Detail of each action or decision was given in respective section of the Guidelines, such as *Initial Review* was discussed in detail in section 3.3.1 of the NORM Guidelines.

As shown in Fig. 1, whenever a NORM Management, Dose Management or Radiation Protection Management Program has been implemented, a *Periodic Review* is needed. The review is to determine if there have been changes to the system that may affect the radiation dose, to monitor the effectiveness of the NORM program and to determine if modifications are required.

This management strategy is driven by decision-points tied to incremental effective dose projections. It works well for most NORMs but not for radon, for two reasons. It is not feasible to quantify incremental radon generated or enhanced by a practice, and the dose from radon at the national guideline level would push the total projected effective dose over the threshold for “NORM Management” in many NORM workplaces (see Table 1), placing an unfair burden on these employers.

Current radon management and associated radon doses are given in Table 2. In the dose calculation, the UNSCEAR radon dose conversion factor of 9 nSv/(h Bq m^− 3^) (UNSCEAR 2020) is used, and an equilibrium factor of 0.4 for radon-222 and its progeny and 2000 h per year exposure duration are assumed.

**Table 2:** Radon management and associated radon effective doses in current Canadian NORM guidelines

Annual average concentration	Annual radon effective dose (mSv/a)
> 800 Bq/m^3^	> 5.8
200–800 Bq/m^3^	1.4–5.8
< 200 Bq/m^3^	< 1.4

The current Canadian NORM Guidelines (Health Canada 2011) recommend radon levels in all workplaces be initially assessed against a radon gas concentration of 200 Bq/m^3^ which, using the UNSCEAR radon dose conversion factor and rounding down, corresponds to about 1 mSv/a. If the average concentration is below 200 Bq/m^3^, no restrictions are required in the workplace even though, mathematically, there appears to be a significant potential for the effective dose to workers to exceed the Dose Management threshold of 1 mSv/a. Not surprisingly, users of the Guidelines find this confusing.

Because of this and other lessons learned through years of questions about how to apply the current NORM Guidelines, we propose explicitly decoupling the decision thresholds for NORM Management (excluding radon) and Radon Management so that the two are considered separately. Further, we propose that the threshold and strategy for occupational radon exposure management in Canada be based on average annual activity concentrations, with no requirement for dose calculations. Proposed application in the updated Canadian NORM Guidelines is described in the next section.

## Proposed radon management in NORM workplaces

A separate, stand-alone Radon Management threshold of 200 Bq/m^3^ is proposed. Measurements should be based on long-term monitoring or testing (a long-term testing normally > 90d). This is not derived from an occupational dose limit or reference level but, rather, is chosen for consistency with Canada’s national radon guidance, which encourages action to reduce exposure when the average annual radon concentration exceeds 200 Bq/m^3^, based on long-term test results, in the normal occupancy area indoors (Health Canada 2007). For the purposes of the NORM Guidelines, exceeding the Radon Management threshold in some areas within a workplace could require either a Radon Management strategy or a Radon Remediation strategy for the area, depending on the distribution of radon within the workplace and working hours in specific locations.

- A Radon Management strategy would be appropriate for situations where radon concentrations are high only in areas with low occupancy. In this case, no intervention would be required for work areas with relatively high ambient radon concentrations as long as workers use them only for short periods of time; specifically, as long as the radon concentration averaged over the a full-time occupancy rate for a full working year (2000 h) does not exceed 200 Bq/m^3^.
- A Radon Remediation strategy would be required for areas where the Radon Management criterion cannot be achieved. It would involve corrective actions, such as increasing ventilation, installing a radon mitigation system, or placing limits on working hours, as needed.

**Fig. 2:**
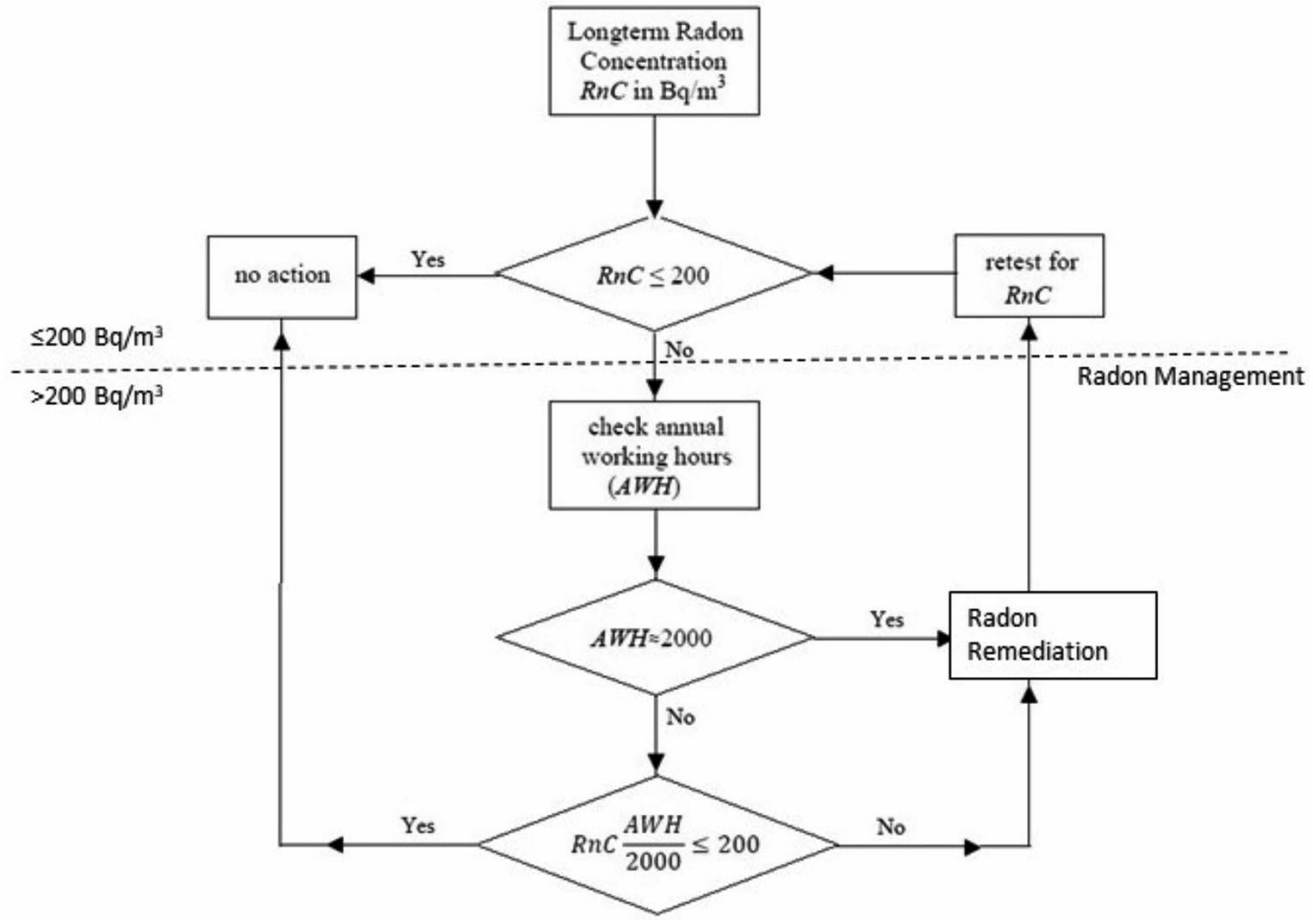
Radon management flowchart

Figure 2 describes this approach. Based on long-term measurements, if the radon concentration, *RnC*, throughout a NORM workplace is less than 200 Bq/m^3^, no action is needed. If the Radon Management threshold is exceeded in some areas, then occupancy rates, i.e. actual annual working hours (*AWH*) in those areas, should be considered when determining whether Radon Management or Radon Remediation is required. *AWH* should realistically represent the most exposed workers and so should correspond to the highest individual occupancy rate. If *AWH* approaches 2000 h per year, corrective actions will need to be taken to reduce radon concentration and resulting annual exposures. However, if workers spend only a few hours per week in an area (*AWH* significantly less than 2000), such as in the treatment room of a waste water treatment facility, special locations in a fish hatchery or some underground storage facilities, we propose that the annual average radon concentration during effective working hours, *RnC·AWH*/*2000*, be considered and compared to the value of the Radon Management threshold in order to determine whether Radon Management or Radon Remediation is required.

For example, consider a water treatment room where the radon concentration is 1000 Bq/m^3^. If workers spend no more than one hour per day in that room (about 230 h per year), the annual exposure level is *1000 × 230/2000 = 115 Bq/m*^*3*^, which is under the threshold of 200 Bq/m^3^. In this case, Radon Mitigation actions are not required; however, a periodic review is recommended. The review is to determine if there have been changes to the workplace and to determine if modifications are needed.

This approach is not intended to discourage employers from voluntarily taking steps to reduce ambient radon concentrations even when they are below the Radon Management threshold. Radon exposure carries some risk at any level and efforts should always be made to keep doses as low as reasonably achievable, with social and economic factors taken into consideration.

### Discussion and conclusions

It is well known that long-term exposure to elevated radon increases the risk of developing lung cancer, and managing exposure to radon is important in all closed environments. It can be particularly important in NORM workplaces such as non-uranium mines, water treatment facility, and fish hatcheries, where radon (and progeny) is very likely the main contributor to occupational dose.

Because radiation doses from NORM (other than radon) tend to be quite low in NORM workplaces, radiation protection is often assigned to all-hazards occupational health and safety specialists as part of a broad portfolio of risks that need to be managed. This means that guidance should be straightforward to understand, to explain, and to implement. For this reason, Canada is proposing to more closely align radon action thresholds in NORM workplaces with national guidelines for public exposure rather than, for example, limits for nuclear workers. The Radon Management threshold is expressed as an activity concentration and does not require the application of dose calculations. Furthermore, the fundamental radiation protection principle of limiting time of exposure is explicitly integrated into the decision-making process.

For practical use and reduction of management burden, we suggest that radon management be simply based on annual average radon activity concentration with consideration of actual annual working hours in a given workplace. If average annual concentration in a given area, when adjusted for occupancy, is not higher than 200 Bq/m^3^, no intervention is required unless occupancy rates or radon concentrations change. This approach is not inconsistent with ICRP’s recommendations for protection of workers (ICRP 2014) which relies on a derived concentration that at which doses will not exceed 10 mSv/a. For reasons of practicality and consistency, the value of the Radon Management threshold is the same as the derived concentration used as an action level for dwellings and for adventitious radon exposures in workplaces and public spaces. Given the nature of NORM workplaces in Canada and our success at reducing radon exposures in uranium mines, we believe that this level of protection is reasonable and, in fact, easily achievable in most cases.
